# An improved 2-level MPPT scheme for photovoltaic systems using a novel high-frequency learning based adjustable gain-MRAC controller

**DOI:** 10.1038/s41598-021-02586-4

**Published:** 2021-11-30

**Authors:** Pankaj Sahu, Rajiv Dey

**Affiliations:** SoET, BML Munjal University, Gurugram, India

**Keywords:** Electrical and electronic engineering, Mathematics and computing

## Abstract

Under rapidly changing environmental conditions, the model reference adaptive control (MRAC) based MPPT schemes need high adaptation gain to achieve fast convergence and guaranteed transient performance. The high adaptation gain causes high-frequency oscillations in the control signals resulting in numerical instability and inefficient operation. This paper proposes a novel high-frequency learning-based adjustable gain MRAC (HFLAG-MRAC) for a 2-level MPPT control architecture in photovoltaic (PV) systems to ensure maximum power delivery to the load under rapidly changing environmental conditions. In the proposed 2-level MPPT control architecture, the first level is the conventional ripple correlation control (RCC) that yields a steady-state ripple-free optimum duty cycle. The duty cycle obtained from the first level serves as an input to the proposed HFLAG-MRAC in the second level. In the proposed adaptive law, the adaptation gain varies as a function of the high-frequency ripple content of the tracking error. These high-frequency contents are the difference between the tracking error and its low-pass filtered version representing the fluctuations in output due to rapid changes in the environmental conditions. Thus, adjusting the adaptation gain by high-frequency content of the tracking error ensures fast convergence, guaranteed transient performance, and overall system stability without needing high adaptation gain. The adaptive law of the proposed HFLAG-MRAC is derived using the Lyapunov theory. Simulation studies, experimental analysis, and performance comparison with recent similar work validate the effectiveness of the proposed work.

## Introduction

**Figure 1:**
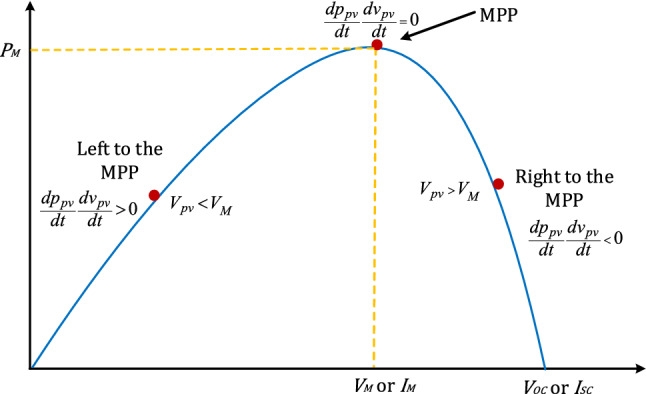
P–V characteristics curve of PV panel.

**Figure 2:**
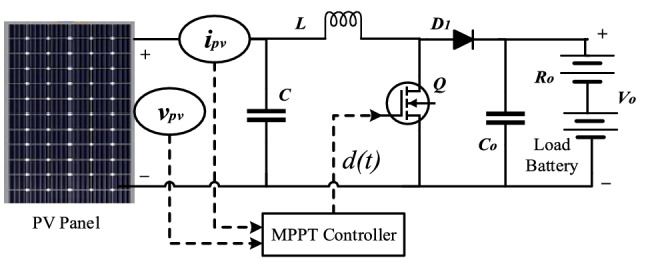
Representation of PV system with single-level MPPT control.

Solar photovoltaic systems (PV) are a significant component to address the issues related to potentially harmful effects on the environment by the elevation in carbon emissions, e.g., global warming. Moreover, a possible reduction in the per-unit power generation cost also concludes that PV systems can fulfil the current growing energy demands. Nowadays, therefore focus of power electronic industries is more on the design, development, and optimum power utilization of the PV system. However, rapid changes in the solar insolation and environmental temperature adversely affect the performance of the PV systems. Due to rapid solar insolation changes, the PV systems cannot endlessly deliver the maximum power to the load^1^. Therefore, algorithms such as maximum power point tracking (MPPT) are needed to rapidly adapt the PV system to environmental changes and deliver optimal power to the load^2^. Commonly, the MPPT control algorithm runs on a microcontroller platform integrated into the power converter whereby controlling its duty cycle optimally, maximum power could be delivered to the load (Fig. 1). A basic structure of PV system with MPPT controller and boost converter is shown in Fig. 2.

A variety of MPPT algorithms mentioned in the literature includes perturb and observe (P&O)^3,4^, hill climbing (HC)^4–6^, fractional techniques^6,7^, soft computing techniques^8,9^, ripple correlation control RCC^10–12^, etc. and their modified versions^3–7,13^. In the first category, such as perturb and observe method, perturbing the array voltage periodically and simultaneously measuring the subsequent updates in the output voltage requires external circuitry. The P&O algorithm is relatively simple and inexpensive. However, its weakness lies in the steady-state performance as instead of continually tracking the MPP it makes the system output oscillate around it. Additionally, the P&O algorithm lacks to adapt itself under varying environmental conditions since it lacks in discriminating the changes in the power level due to the environmental factors and changes in the power level due to the intrinsic perturbations.

The HC algorithm is similar to the P&O algorithm, but they differ in the perturbation parameters. In the P&O algorithm, the array voltage or current is perturbed to achieve MPP. Whereas, in HC duty cycle is perturbed. The P&O, HC methods suffer from a common problem that they need a trade-off between the steady-state and the transient performance. This problem is more severe in the HC algorithm as instead of directly controlling the array voltage the duty cycle is controlled. Additionally, the HC algorithm fails to achieve MPP tracking under changing environmental conditions^3,4^.

The fractional open-circuit voltage (FOCV) algorithm approximates the relationship between the open circuit array voltage $V_{oc}$ and the array voltage at which the maximum power is achieved $V_{M}$^6,7^. Similar to the P&O algorithm, the FOCV is easy to implement. However, the approximated relationship between the $V_{oc}$ and $V_{M}$ is just an assumption and therefore, FOCV is not a true MPP tracker. Similar to FOCV, the fractional short-circuit current algorithm utilizes the short circuit array current $I_{sc}$ to track MPP. Soft computing techniques such as fuzzy logic and neural network-based algorithms perform well under rapid changes in environmental conditions. However, the algorithms are complex and somewhat difficult to implement^8^.

The RCC method utilizes the inherent ripples associated with the DC–DC power converters to track the MPP. It uses the voltage, current, or power ripples and correlates with the switching function (duty cycle) to deliver the maximum available power to the load^10–13^. However, to counter the effects of rapidly changing environmental conditions, this approach suffers from transient oscillations at the system’s output voltage after each update of the duty cycle.

**Figure 3:**
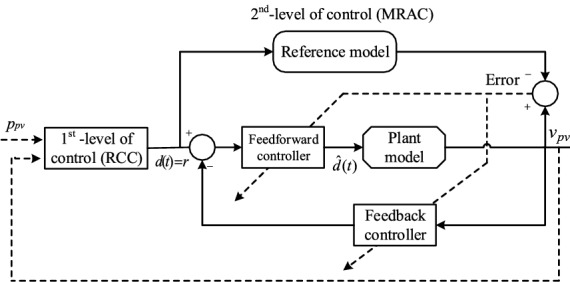
Representation of 2-level MPPT MRAC control architecture^2^.

To avoid such significant fluctuations in the PV system output voltage in the transient phase, Khanna et al.^2^ proposed a 2-level MPPT architecture based on adaptive control shown in Fig. 3. In the proposed algorithm, the first level consists of the RCC unit that takes input as array voltage, power and calculates the optimum duty cycle to deliver maximum available power to the load. The updated duty cycle calculated by the RCC is feed as an input to the second stage, i.e., model reference adaptive control (MRAC), to handle the faster dynamics of the entire PV system and eliminate the possible oscillations in the transient phase. To avoid any transient oscillations after each update in the duty cycle at the PV system's output, a critically damped reference model is chosen.

The MRAC in 2-level MPPT ensures the improved transient performance of the PV system under rapid changes in the environmental conditions^2,14^. However, achieving improved transient performance with fast convergence in MRAC, high adaptation gain is needed. The high adaptation gain may cause high-frequency oscillations in the control signal and make the differential equation of the adaptive law stiff, causing numerical instability even in a high-speed computer. Additionally, the high adaptation gain may lower the time delay margin to a certain extent and puts a constraint on the closed-loop PV system. This imposes small control law calculating time and small-signal transmitting time; both of these are difficult to forecast accurately^15,16^. Therefore, it is apparent to improve the transient performance without the need for high adaptation gain. Similar work can be found in^14^. To the best of our knowledge no work has been done in this area dealing with such kind of problem. This paper is mainly aimed at developing the modified MRAC control architecture.

**Figure 4:**
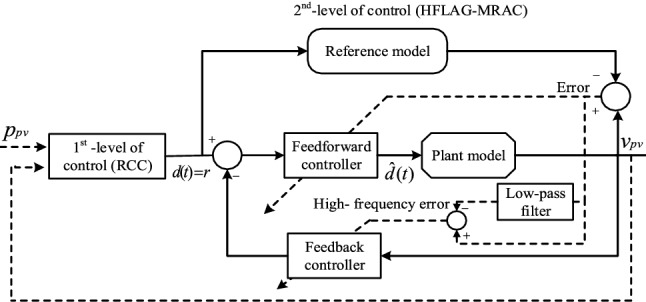
Representation of the proposed 2-level MPPT MRAC control architecture.

Inspired from the previous work^2^, a new adaptive control strategy for a 2-level MPPT architecture (HFLAG-MRAC shown in Fig. 4) is proposed in this paper aiming to achieve fast convergence with guaranteed transient performance. The novelty of this work is to utilize the derived high-frequency (ripple) content of the tracking error to optimally adjust the adaptation gain of the adaptive law. This high frequency error is computed as the difference between the tracking error and its low-pass filter version and truly represents the variations in environmental parameters. Therefore, under frequent changes in environmental conditions the adaptive law updates much efficiently to rapidly converge the tracking error to zero ensuring fast convergence and guaranteed transient performance. Additionally, the proposed HFLAG-MRAC controller ensures the absence of high-frequency oscillations in control signal $\hat{d}\left( t \right)$.

Similar to Khanna et al.^2^, the first level in the proposed 2-level MPPT architecture is the RCC. The RCC takes the input from the PV array voltage $v_{pv}$ and power $p_{pv}$ and calculates the optimal duty cycle $d\left( t \right)$ in steady-state. The second level is the proposed HFLAG-MRAC that utilizes the optimum steady state duty cycle $d\left( t \right) = r$ computed by the RCC as input and control faster dynamics of the DC–DC converter under variable environmental conditions.

The organization of the paper is as follows: background of the converter dynamics and the MPPT problem in the context of PV systems has been discussed in “Background and system description” section. In “Proposed methodology” section describes the proposed 2-level MPPT control architecture along with the review of RCC followed by theoretical simulation model in “Simulation model of proposed 2-level MPPT control” section and simulation results in “Simulation results and discussions” section followed by experimental results in “Experimental results” section.

## Background and system description

### MPPT in PV systems

In a basic PV system, voltage or current of the solar panel is regulated with the help of a dc-dc converter interfaced with an MPPT controller to obtain the maximum power as shown in Fig. 2. The MPPT controller takes voltage $v_{pv} { }$ and current $i_{pv}$ from the PV panel through sensors and yields duty cycle $d\left( t \right)$ to control the switching transistor ${ }Q$. This PV array voltage and current consists of both DC and ripple terms. The role of the MPPT control algorithm is to extract maximum available solar power and deliver it to the load, such that the output array voltage $V_{pv}$ tracks $V_{M}$ or and output array current $I_{pv}$ tracks $I_{M}$ as shown in Figs. 1 and 6.

For a basic PV system with single level MPPT control, the steady-state relation between the PV array voltage $v_{pv} ,$ PV array current $i_{pv}$ and the duty cycle $d$ of the switching transistor $Q$ can be expressed as^4^;

1 $$v_{pv} = i_{pv} { }R_{o} \left( {1 - d} \right)^{2} ,$$

with, $v_{pv} = V_{pv} + \hat{v}_{pv}$ and $i_{pv} = I_{pv} + \hat{i}_{pv}$,where $V_{pv}$, $I_{pv}$ are the average DC terms of PV array voltage and current, $\hat{v}_{pv} ,$ $\hat{i}_{pv}$ are the ripple terms, and $R_{o}$ is the load resistance.

### Small-signal modelling of PV system with power-conversion stage

**Figure 5:**
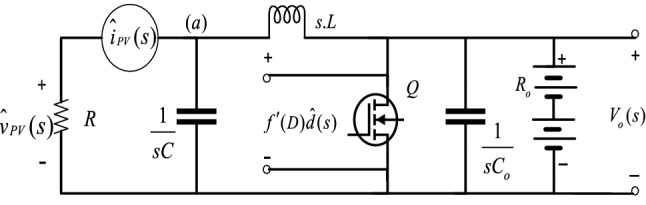
Small signal model of PV power converter stage.

The basic relation between the converter’s duty cycle and the array voltage is provided in (1) for steady-state. However, to improve the transient response, the dynamics between the duty cycle and array voltage must be considered in MPPT control. To simply the analysis of system’s transient response a small signal equivalent of Fig. 2 is considered which is shown in Fig. 5, in a similar way as^2,3,14^.

In Fig. 5 the resistance $R$ represents PV array with small signal array voltage $\hat{v}_{pv} \left( s \right)$ and small signal array current $\hat{i}_{pv} \left( s \right)$. Now, Ignoring the dynamics of the load i.e. battery, the transfer function from the duty cycle to the array voltage in small signal operation is derived about an operating point. By applying KCL at node (a), the relationship between array voltage $\hat{v}_{pv} { }$ with the small signal variation around the converter’s duty cycle $\hat{d}$, can be obtained in S-domain as

$$\frac{{\hat{v}_{pv} \left( s \right)}}{R} + \hat{v}_{pv} \left( s \right)Cs = \frac{{f^{\prime}\left( D \right)\hat{d}\left( s \right) - \hat{v}_{pv} \left( s \right)}}{sL}.$$

Upon rearranging the above expression, we get

2 $$\frac{{\hat{v}_{pv} \left( s \right)}}{{\hat{d}\left( s \right)}} = \frac{{f^{\prime}\left( D \right)}}{{LCs^{2} + \frac{{\text{L}}}{R}s + 1}},$$

where $\hat{v}_{pv} \left( s \right)$ and $\hat{d}\left( s \right)$ are the Laplace transforms of $\hat{v}_{pv} \left( t \right)$ and $\hat{d}\left( t \right)$ respectively. The function $f\left( D \right){ }$ is the relationship between the operating duty cycle $D$, steady-state DC PV array voltage ${ }V_{pv}$, and output array voltage $V_{o} { }$ of the boost converter and can be given as^4^

3 $$f\left( D \right) = V_{pv} = \left( {1 - D} \right)V_{o} .$$

Taking the first derivative of (3) with respect to duty cycle $D$, we get

4 $$f^{\prime}\left( D \right) = - V_{o} { },$$

substituting the value of $f^{\prime}\left( D \right)$ from (4) in the (2), we get

5 $$\frac{{\hat{v}_{pv} \left( s \right)}}{{\hat{d}\left( s \right)}} = \frac{{\frac{{ - V_{o} }}{LC}}}{{s^{2} + \frac{1}{RC}s + \frac{1}{LC}}}.$$

**Figure 6:**
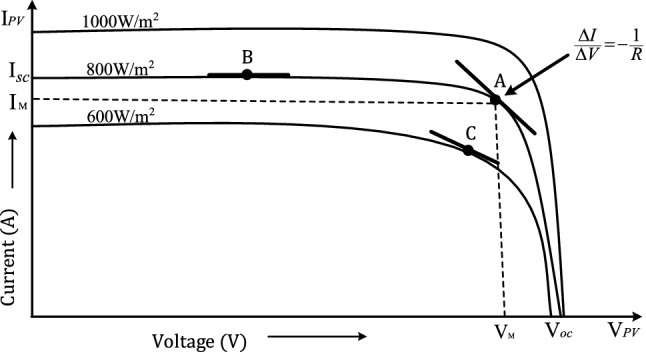
I-V characteristics of a solar panel with varying *R*^2^.

Equation (5) represents the second order linear model of Fig. 4 around MPP, in which $C$ and $L$ are known, with unknown $R$. Under variable environmental conditions, operating point of the systems gets changed, specifically the value of $R$ varies in accordance with the changing solar insolation. The value of $R$ can be determined from I-V curve as the magnitude of the inverse slope of the line tangential to point A as shown in Fig. 6^2^.

6 $$\frac{1}{R} \approx - \frac{\Delta I}{{\Delta V}}.$$

The Fig. 6 shows the I–V characteristics of a solar panel for various solar insolation levels. The MPP for the curve related to 800 W/m^2^ is notated by the point A ($V_{M} ,I_{M}$). Under constant solar insolation, if the operating point shifts from point A to point B, the value of R gets changed. Again, at solar insolation of 600 W/m^2^ if the operating point shifts from point A to point C, a new value for R is found. Therefore, even for a new MPP value, there is no guarantee that the operating R will be equal to the previous optimal R. Moreover, the optimal operating R to exhibit a critically damped system characteristic is also not guaranteed^2^. To illustrate the effect of R on the system performance we compare the denominator of (5) with the standard 2nd-order characteristics equation $s^{2} + 2\zeta \omega_{n} + \omega_{n}^{2}$ we get

$$\omega_{n} = \frac{1}{{\sqrt {LC} }}\;\;{\text{and}}\;\;\zeta = \frac{1}{2R}\sqrt{\frac{L}{C}} ,$$

where $\omega_{n}$ is the natural frequency rad/sec, $\zeta$ is damping ratio.

From the above expressions of $\omega_{n}$ and $\zeta$ it is clear that damping ratio is inversely proportional to *R*. Therefore, any variations in the solar irradiance levels and temperature will cause the operating point to vary and the values of *R* for which $\zeta < 1$, the system will exhibit under damped behavior which cause oscillations in the step response.

In this paper, an improved 2-level MPPT control scheme has been proposed to effectively track the MPP under the changing environmental conditions. The first level of control is the RCC, which is used to bring the operating point to the optimal value of *R*, by performing correlation between high frequency (ripple) content and switching function^11–13^. The second level of control is the novel HFLAG-MRAC controller which regulates the converter dynamics such that the optimal *R* also delivers a critically damped system response under changing environmental conditions. The adaptation gain of the proposed HFLAG-MRAC, is adjusted in accordance to the high-frequency (ripple) content of the tracking error, in a similar approach as RCC. These high-frequency (ripple) content of the tracking error truly represents the variations in input parameters. Therefore, the adaptation gain of the proposed HFLAG-MRAC is adjusted more effectively to handle the effect of changing environmental conditions on the PV system’s output voltage. Thus, ensures fast convergence, improve transient performance without the need of high adaptation gain along with the assurance of absenteeism of high-frequency oscillations in control signal. The detailed methodology of the proposed work has been provided in sub-sequent section.

## Proposed methodology

**Figure 7:**
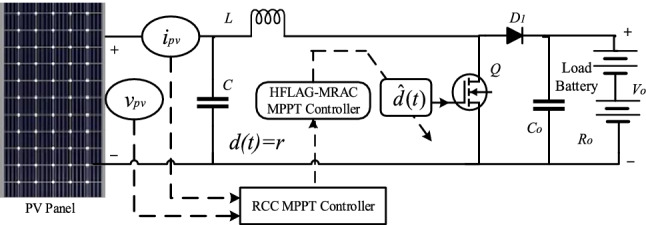
Representation of PV system with proposed 2-level MPPT control architecture.

In Fig. 7, it can be seen that the proposed 2-level MPPT control approach has the RCC unit as first level of control. The RCC unit is used to handle the slower dynamics of the converter in response to the changing solar insolation and calculate the optimum duty cycle in steady state. The output of the RCC unit acts as an input for the second level of the MPPT control. In the second level, the proposed HFLAG-MRAC control unit regulates the faster dynamics of the converter in response to the duty cycle $d\left( t \right) = r$ calculated from the first level and prevents transient oscillations in array voltage after variations in solar insolation. In the proposed 2-level MPPT, the RCC unit is used to handle changes in solar insolation, and the tuning of RCC must be adequately fast enough to capture the changes in solar insolation. Therefore, RCC must have a smaller time constant compared to the dynamics of insolation changes^2^. For a decoupled analysis in 2-level MPPT control approach the time constant of the 1st-level must be smaller than the dynamics of environmental changes and the time constant of the 2nd-level of control must be less than the 1st-level. The significant difference between these time constants gives liberty to decouple the analyses for 1st and 2nd levels; this, simplifies the overall MPPT control design^2,14^.

In comparison to the RCC, the proposed HFLAG-MRAC unit is accountable for maintaining optimal damping characteristics of the converter whose time-constant is very less than the environmental changes. Therefore, the adaptation scheme of the proposed HFLAG -MRAC level must be adequately fast enough to respond with the rapid variations in the operating point of the converter and the also to the rapid change in duty-cycle of RCC, under fast changing environment conditions. The HFLAG -MRAC handles the faster dynamics of the converter to counter the variations in solar insolation and duty cycle input from the RCC unit and adjusts its gain as a function of high-frequency content of the tracking error. Thus, guarantees improved transient response with the absence of high-frequency oscillations in control signal of the controller.

To develop the control law for the proposed HFLAG-MRAC unit a second order linear plant model of PV system is considered, as given in (5). This important relation provides, the plant and reference model required for state-space analysis, which is necessary to derive update laws for HFLAG-MRAC. This work mainly focuses on developing an improved 2-level MPPT scheme with a novel 2nd-level of MPPT control i.e., HFLAG-MRAC control algorithm for improved transient performance.

In the upcoming “Ripple correlation control” section, a detailed review of conventional RCC technique has been discussed for steady-state duty cycle calculation. Further, in order to derive error dependent adjustable gain update laws for the proposed HFLAG-MRAC unit, necessary mathematical modelling and formulations, along with state-space analysis of MRAC has been discussed in “Basic formulations used for HFLAG-MRAC design” section. In “The proposed HFLAG-MRAC controller” section provides the mathematical modelling of the proposed HFLAG-MRAC, which is the main contribution of this paper.

### Ripple correlation control

RCC, is a nonlinear control approach applicable to power electronic circuits^10–12^. The objective of this RCC unit is find out the ripple-free optimum duty cycle in steady-state. RCC takes voltage and current inputs from the solar panel and calculates ripple content of power and voltage. Then, these ripples are correlated with the switching function to calculate optimal duty cycle. In the proposed 2-level architecture RCC at 1st-level of control, is used to calculate the ripple free optimal duty cycle $d\left( t \right)$ to deliver maximum power in steady-state^2^. This calculated duty cycle $d\left( t \right)$ serves as an input for the proposed novel HFLAG-MRAC unit.

**Figure 8:**
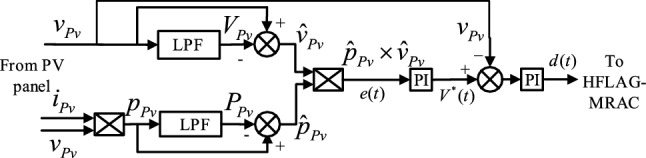
Architecture of 1st-level of the proposed 2-level MPPT control (RCC)^11^.

Simple circuit implementation, no latency in computation/simulation, and no necessity of external perturbation (as in P&O and IC), to reach MPP, are the major advantages of RCC technique. Additionally, it converges asymptotically to the object, which is MPP in this work with a facility to tune its convergence rate by the controller gain, makes this technique more attractive. Architecture of RCC^11^ has been shown in Fig. 8. The error voltage $e\left( t \right)$ is the product of power and voltage ripples, used to obtain a reference signal $V^{*} \left( t \right)$ after processing from first proportional controllers PI, which is passed through second PI after subtracted from array voltage $v_{pv} .$ The RCC system utilizes high-frequency or ripple content of PV array voltage and power, to yield optimal ripple free duty cycle $d(t$) for HFLAG-MRAC unit, which is obtained through subtraction of input from low-pass filters (LPF) version of input. The proposed HFLAG-MRAC approach has been inspired by the same concept to utilize the high-frequency content of tracking error to adjust the adaptation gain.

The RCC utilizes inherent ripples of the system and through the correlation of the time-based derivative of voltage and power it tries to identify whether this correlation is greater than zero i.e., to the left of the MPP, or less than zero i.e., to the right of the MPP, or exactly zero” i.e., equal to MPP^10–12^, which is also shown in Fig. 1 for representation.

7 $$\begin{aligned} { }\frac{{dp_{pv} }}{dt}\frac{{dv_{pv} }}{dt} & > 0{ }\;when\;V_{PV} < V_{M} , \\ \frac{{{ }dp_{pv} }}{dt}\frac{{dv_{pv} }}{dt} & < 0\;when\;V_{PV} < V_{M} , \\ { }\frac{{dp_{pv} }}{dt}\frac{{dv_{pv} }}{dt} & = 0\;when\;V_{PV} = V_{M} . \\ \end{aligned}$$

From the above equation, the following control law can be derived as,

8 $$\frac{{{ }dd\left( t \right)}}{dt} = k\frac{{{ }dp_{pv} }}{dt}\frac{{dv_{pv} }}{dt},$$

where $k$ is the negative gain constant. The summary of above control law mentioned in (8) can be described as provided in Table 1.

**Table 1:** Summary of RCC control laws, (7) and (8).

If $v_{pv}$	Effects in-$p_{pv}$	Operating point is at	Action for-$d\left( t \right)$	Results	Average value of error
Increases	Increases	Left to the MPP	Decreased	Increase in $v_{pv}$	Positive
Increases	Decreases	Right to the MPP	Increased	Decrease in $v_{pv}$	Negative
$= V_{M}$	$= P_{M}$	At MPP	No change	No change	0

From the Table 1, it can be interpreted that the objective of RCC is to make the time-based derivative of $d\left( t \right)$ equals to 0, so that the system remains at MPP and yield maximum power. Since, RCC is well studied in the literature^2,10–12^, and it has been analyzed theoretically and proven mathematically to yield the optimal duty cycle to extract the maximum obtainable power in the steady-state. Therefore, only a brief analysis of RCC has been presented in this work. In the next sub-section, detailed modelling of the proposed HFLAG-MRAC along with all necessary formulations have been presented.

### Basic formulations used for HFLAG-MRAC design

**Figure 9:**
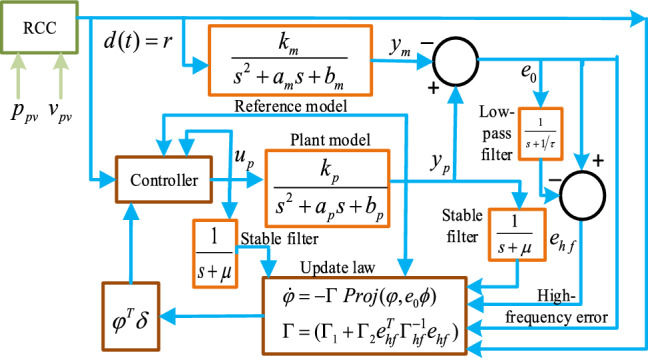
Architecture of 2nd-level of MPPT control (Novel HFLAG-MRAC controller).

The architecture of the proposed 2nd-level of control i.e., HFLAG-MRAC is shown in Fig. 9. The proposed HFLAG-MRAC acquires its reference signal $d(t = r)$ from the RCC unit. The aim of the HFLAG-MRAC design is to achieve improved transient performance in MPPT, by handling the faster converter dynamics of the non-linear PV systems under changing environment conditions, without the need for high-static adaptation gain. In the first level of the proposed MPPT control the RCC, utilizes the high frequency content and correlate it with the switching function to affect control^11^.

In a similar manner as RCC, the proposed HFLAG-MRAC utilizes the high-frequency (ripple) content of the tracking error to adjust its gain in response to the variations in inputs and yields critically-damped PV system characteristics under rapidly changing conditions. In PV systems, these high-frequency (ripple) contents of the tracking error are the actual representation of rapid variations appearing in input parameters due to change in solar insolation, temperature, and thus, in duty-cycle $d\left( t \right)$. Therefore, for an accurate modelling and MPPT control, the gain of the second-level of control must be adjusted in accordance with the high-frequency (ripple) content of the tracking error, instead of overall tracking error.

In order to derive the proposed high-frequency error dependent adjustable gain update-laws for the MRAC controller, the necessary detailed modelling of MRAC^17,18^, has been discussed first in this sub-section and then main contribution of this paper is presented in “The proposed HFLAG-MRAC controller” section, as Theorem 1, along with the proof.

#### Reference and plant model

The basic idea of designing MRAC system is that the plant model output should always tracks the reference model output^17,18^. According to (5), the plant model for the proposed HFLAG-MRAC with positive coefficients is considered as^2,14^

9 $$W_{p} \left( s \right) = \frac{{y_{p} \left( s \right)}}{{u_{p} \left( s \right)}} = \frac{{k_{p} }}{{s^{2} + a_{p} s + b_{p} }},$$

where the values of ${ }k_{p}$, $a_{p}$, and $b_{p}$ can be interpreted from (5). The reference model is given by

10 $$W_{m} \left( s \right) = \frac{{y_{m} \left( s \right)}}{{u_{m} \left( s \right)}} = \frac{{k_{m} }}{{s^{2} + a_{m} s + b_{m} }},$$

where $k_{m}$ is a positive gain and $a_{m}$ and $b_{m}$ should be chosen such that ζ = 1. The control objective is to develop a suitable control law $u_{p} \left( s \right)$ such that $y_{p} \left( s \right)$ always follows $y_{m} \left( s \right)$.

In order to find the suitable error-dependent adjustable gain strategy for improved transient response in 2-level MPPT control, the controller structure and state-space analysis has been discussed in next sub-sections.

#### Controller structure

To attain the desired control objectives, the controller structure has been considered similar as^2,14,17,18^. For bounded reference input the control law $u_{p} { }$ is considered as

11 $$u_{p} = \varphi_{0} r + \varphi_{1} \frac{1}{s + \mu }u_{p} + \varphi_{2} \frac{1}{s + \mu }y_{p} + \varphi_{3} = \varphi^{T} \delta ,$$

where $\varphi = \left[ {\varphi_{0} ,{ }\varphi_{1} ,{ }\varphi_{2} ,{ }\varphi_{3} } \right]^{T}$ is the parameter estimation vector of the controller, and $\delta$ is defined as $\delta = [r,\delta_{1} ,{ }\delta_{2} ,{ }y_{p} ]^{T}$ with $\delta_{1} = \frac{1}{s + \mu }u_{p} ,$ and $\delta_{2} = \frac{1}{s + \mu }y_{p} ,$ and $\frac{1}{s + \mu }$ is a stable low-pass filter with $\mu > 0$, obtained through genetic algorithms^19^. $\delta_{1}$ and $\delta_{2}$ can be resolute by (12) as

12 $$\begin{aligned} \dot{\delta }_{1} & = - \mu \delta_{1} + u_{p} \\ \dot{\delta }_{2} & = - \mu \delta_{2} + y_{p} \\ \end{aligned}$$

As per^17^, the controller (11) is adequate to attain the desired control objective. It is possible to make $y_{p} \left( s \right)/r\left( s \right)$ equals to ${ }y_{m} \left( s \right)/r\left( s \right)$ when $\varphi$ equals $\varphi^{*} = \left[ {\varphi_{0}^{*} ,{ }\varphi_{1}^{*} ,{ }\varphi_{2}^{*} ,{ }\varphi_{3}^{*} } \right]^{T} { }$ with

13 $$\begin{aligned} \varphi_{0}^{*} & = \frac{{k_{m} }}{{k_{p} }}, \\ \varphi_{1}^{*} & = a_{p} - a_{m} , \\ \varphi_{2}^{*} & = \frac{{\left( {a_{p} - a_{m} } \right)\left( { - \mu + \mu a_{p} - b_{p} } \right)}}{{k_{p} }}, \\ \varphi_{3}^{*} & = \frac{{b_{p} - b_{m} + \left( {a_{p} - a_{m} } \right)\left( { - \mu + \mu a_{p} - b_{p} } \right)}}{{k_{p} }}. \\ \end{aligned}$$

#### State-space representation

Assuming {$A_{p}$, ${ }B_{p}$, ${ }C_{p}$} be a minimal realization of the controlled plant $W_{p}$(s), as follows

14 $$\begin{aligned} \dot{x}_{p} & = A_{p} x_{p} + B_{p} u_{p} , \\ y_{p} & = C_{p} x_{p} , \\ \end{aligned}$$

where $x_{p}$ is a 2-D state vector. Considering the controller dynamics in (11), (12), the closed-loop system with plant and its controller can be written as

15 $$\begin{aligned} \dot{x}_{pe} & = A_{pe} x_{pe} + B_{pe} \varphi_{0}^{*} r + B_{pe} \left( {u_{p} - \varphi^{{{*}T}} \delta } \right), \\ y_{p} & = C_{pe} x_{pe} , \\ \end{aligned}$$

where $x_{pe}$ is the extended error vector defined as.$x_{pe} = \left[ {x_{{\text{p}}}^{{\text{T}}} ,{ }\delta_{1} ,{ }\delta_{2} } \right]^{{\text{T}}}$, $\varphi^{*}$ is resolute by (13) and matrices ${ }A_{{{\text{pe}}}} ,$ $B_{{{\text{pe}}}}$, and $C_{{{\text{pe}}}}$ are defined as

$$A_{pe} \equiv \left[ {\begin{array}{*{20}c} {A_{p} + \varphi_{3}^{*} B_{p} C_{p} } & {\varphi_{1}^{*} B_{p} } & {\varphi_{2}^{*} B_{p} } \\ {\varphi_{3}^{*} B_{p} } & { - \mu + \varphi_{1}^{*} } & {\varphi_{2}^{*} } \\ {C_{p} } & 0 & { - \mu } \\ \end{array} } \right]$$

$$B_{pe} \equiv \left[ {\begin{array}{*{20}c} {B_{p} } \\ 1 \\ 0 \\ \end{array} } \right]$$

$$C_{pe} \equiv \left[ {\begin{array}{*{20}c} {C_{p} } & 0 & 0 \\ \end{array} } \right]$$

Now, with $u_{{\text{p}}} = \varphi^{{{*}T}} \delta$, (15) becomes

16 $$\begin{aligned} \dot{x}_{pe} & = A_{pe} x_{pe} + B_{pe} \varphi_{0}^{*} r \\ y_{p} & = C_{pe} x_{pe} \\ \end{aligned}$$

Therefore, it can be inferred that $y_{p} \left( s \right)/r\left( s \right) = y_{m} \left( s \right)/r\left( s \right)$. In turn, {$A_{{{\text{pe}}}} ,$ $\varphi_{0}^{*} B_{{{\text{pe}}}} ,C_{{{\text{pe}}}} { }\}$ must be a realization of the reference model and can be given as

17 $$\begin{aligned} \dot{x}_{me} & = A_{pe} x_{me} + B_{pe} \varphi_{0}^{*} r \\ y_{m} & = C_{pe} x_{me} , \\ \end{aligned}$$

where $x_{me}$ is the 4-D state vector of the aforesaid realization with $A_{{pe{ }}}$ being asymptotically stable.

#### Error dynamics

The error dynamics can be obtained by subtracting (17) from (15)

18 $$\begin{aligned} \dot{e} & = A_{pe} e + B_{pe} \left( {u_{p} - \varphi^{{{*}T}} \delta } \right) \\ & = A_{pe} e + B_{pe} \tilde{\varphi }^{{\text{T}}} \delta , \\ e_{0} & = C_{pe} e, \\ \end{aligned}$$

where $e \equiv x_{pe} - x_{me}$ is the state error, $e_{0} \equiv y_{p} - y_{m}$ is the output error, and $\tilde{\varphi } = \varphi - \varphi^{*}$ is the estimation error.

To determining the adaptive law of the proposed controller utilizing Lyapunov function, the state error-equation’s input–output transfer function must be Strictly Positive Real (SPR)^2^. Whereas, the transfer function of the realization {$A_{{{\text{pe}}}} ,B_{{{\text{pe}}}} ,C_{{{\text{pe}}}} { }\}$ in (14) is not SPR, and from (19) $C_{pe} \left( {sI - A_{pe} } \right)^{ - 1} B_{pe}$ equals $W_{m} \left( s \right)/{ }\varphi_{0}^{*}$. According to the (10) the relative degree of $W_{m} \left( s \right)$ is 2, which implies that $W_{m} \left( s \right)/{ }\varphi_{0}^{*}$ is not SPR^17,18^. To overcome this exertion the identity $\left( {s + \alpha } \right)\left( {s + \alpha } \right)^{ - 1} { }$ = 1 for some α > 0 is used. Rewriting (18) using this identity we get

19 $$\begin{aligned} \dot{e} & = A_{pe} e + B_{pe} \left\{ {\left( {s + \alpha } \right)\left( {u_{\alpha } - \varphi^{{{*}T}} {\Phi }} \right)} \right\} \\ & = A_{pe} e + B_{pe} \left\{ {\left( {s + \alpha } \right)\tilde{\varphi }^{T} {\Phi }} \right\} \\ e_{0} & = C_{pe} e, \\ \end{aligned}$$

where $u_{\alpha } \equiv \frac{1}{s + \alpha }u_{p}$ and ${\Phi } = \frac{1}{s + \alpha }\delta .$ Also, $s + \alpha$ will augment the degree of numerator so that the relative degree of the transfer function becomes 1. Since $u_{\alpha } = \varphi^{{\text{T}}} {\Phi },{ }$ the control law can be written as

20 $$\begin{aligned} u_{p} & = \left( {s + \alpha } \right)u_{\alpha } = \dot{\varphi }^{{\text{T}}} {\Phi } + \varphi^{T} {\dot{\Phi }} + \alpha \varphi^{T} {\Phi } \\ & { = }\dot{\varphi }^{T} {\Phi } + \varphi^{T} \left( {{\dot{\Phi }} + \alpha {\Phi }} \right). \\ \end{aligned}$$

Upon introducing

21 $$\overline{e} = e - B_{pe} \tilde{\varphi }^{T} {\Phi ,}$$

It can be derived that

22 $$\begin{aligned} \dot{\overline{e}} & = A_{pe} \overline{e} + \left( {A_{pe} B_{pe} + \alpha B_{pe} } \right)\tilde{\varphi }^{T} {\Phi } \\ & = A_{pe} \overline{e} + B_{1} \tilde{\varphi }^{T} {\Phi }, \\ \end{aligned}$$

23 $$\begin{aligned} e_{0} & = C_{pe} \overline{e} + C_{pe} B_{pe} \tilde{\varphi }^{T} {\Phi } \\ & = C_{pe} \overline{e}, \\ \end{aligned}$$

where $B_{1} = A_{pe} B_{pe} + \alpha B_{pe}$ and $C_{pe} B_{pe} = 0$. Since, the relative degree of reference model in (10) is 2, i.e., the leading coefficient for the numerator of $C_{pe} \left( {sI - A_{pe} } \right)^{ - 1} B_{pe}$ is 0. For the new state-error Eq. (22) the transfer function from $\tilde{\varphi }^{T} {\Phi }$ to $e_{0} { }$ must be same as the transfer function in (19). As (23) is equally transformed from (19). Also, from (19) and (23), it can be concluded that $e = \overline{e}$. Therefore, the realization of {$A_{{{\text{pe}}}} ,$ $B_{1} ,C_{{{\text{pe}}}} { }\}$ has the following transfer function

24 $$C_{pe} \left( {sI - A_{pe} } \right)^{ - 1} B_{1} = \frac{{k_{m} }}{{\varphi_{0}^{*} }}{ } \cdot { }\frac{s + \alpha }{{s^{2} + a_{m} s + b_{m} }}.$$

It can be shown that (24) is SPR for any α satisfying, 0 < α < $a_{m}$.

The above formulation of MRAC systems is the pre-requisites, in order to derive high-frequency (or ripple content) error dependent adjustable gain update law for proposed HFLAG-MRAC controller. In the next sub-section, the update law for HFLAG-MRAC is established in Theorem 1.

### The proposed HFLAG-MRAC controller

In this section, the adaptive law for the proposed HFLAG-MRAC has been derived in Theorem 1.

#### **Theorem 1**

*Let* $\Gamma_{1} > 0$, ${\Gamma }_{2} > 0,{ }$ *and* ${\Gamma }_{hf} > 0$ *be constants*, $\varphi \left( t \right) \in {\Omega },{ }\overline{e}_{hf} \left( t \right) \in {\Omega }_{1}$ *and* $e_{0} \left( {\text{t}} \right) \in {\Omega }_{2}$ *(a convex set); then for the linear system given by Eq.* (14) *and controlled by Eqs*. (11)–(13), *the update law for the proposed HFLAG-MRAC can be given as*

25 $$\begin{aligned} \dot{\tilde{\varphi }}{ } & = - {\Gamma }Proj{ }\left( {\varphi ,e_{0} {\Phi }} \right) \\ \varphi \left( 0 \right) & = \varphi_{0} \\ {\Gamma } & = {\Gamma }_{1} + {\Gamma }_{2} { }e_{hf}^{T} {\Gamma }_{lf}^{ - 1} {\text{e}}_{hf} > 0, \\ \end{aligned}$$

*where the* $Proj$ *is the projection operator*^16^ *ensures boundedness of the function and is defined as*

26 $${\text{Proj }}\left( {\varphi ,\psi } \right) = \left\{ {\begin{array}{*{20}l} {0,} \hfill & {\quad if\;\varphi = \varphi_{max} \;and\;\psi > 0} \hfill \\ {0,} \hfill & {\quad if\;\varphi = \varphi_{min} \;and\;\psi < 0} \hfill \\ {\psi ,} \hfill & {\quad otherwise, } \hfill \\ \end{array} } \right.$$

*the following upper bound for the state tracking error* $\overline{e}$*, parameter estimation error* $\tilde{\varphi }$ *and low-pass filtered state error* $\overline{e}_{hf}$ *holds*

27 $$\begin{aligned} \left\| {\overline{e}} \right\|_{\infty } & \le \sqrt {\frac{\chi }{{\lambda_{min} \left( P \right)\left\{ {{\Gamma }_{1} + {\Gamma }_{2} { }\omega^{2} { }\left\| {{\Gamma }_{hf}^{ - 1} } \right\|} \right\}}}} , \\ \left\| {\tilde{\varphi }} \right\|_{\infty } & \le \sqrt \chi ,\;\;{\text{and}}\;\;\left\| {\overline{e}_{hf} } \right\|_{\infty } \le \sqrt \chi , \\ \end{aligned}$$

*where* $$\chi \triangleq { }4_{max} \left\| \varphi \right\|^{2} + 4_{max} \left\| {\overline{e}_{hf} } \right\|^{2} + \frac{{2\lambda_{max} \left( P \right)({\Gamma }_{1} \overline{e}^{T} P\overline{e} + 1)\omega \upsilon }}{{\left\| {{\Gamma }_{hf} } \right\|}}$$.

#### ***Proof***

The proof of theorem 1 can be obtained in a similar way as in^20,21^. For the proof the Lyapunov function can be considered as

28 $$V\left( {\overline{e},\tilde{\varphi }{ },\overline{e}_{hf} } \right) = \left( {{\Gamma }_{1} + {\Gamma }_{2} { }\overline{e}_{hf}^{T} {\Gamma }_{hf}^{ - 1} \overline{e}_{hf} } \right)\overline{e}^{T} P\overline{e} + \tilde{\varphi }^{T} \tilde{\varphi } + { }\overline{e}_{hf}^{T} {\Gamma }_{hf}^{ - 1} { }\overline{e}_{hf} ,$$

where ${\Gamma }_{{1,{ }}} {\Gamma }_{2}$ are symmetric positive definite static gains, and ${\Gamma }_{hf}$ is positive definite high-pass filter gain matrix and its filtered error dynamics is given by

29 $$\dot{\overline{e}}_{hf} = \left( {\dot{\overline{e}} - {\Gamma }_{hf} \overline{e}_{hf} } \right),$$

where $\overline{e}_{hf}$ is the resultant output of the high-pass filter, in s-domain it is given as,

30 $${ }\overline{e}_{hf} = \frac{{s\tau \cdot \overline{e}\left( s \right)}}{\tau s + 1},$$

where $\tau$ is the time constant of the low-pass filter, ${\Gamma }_{hf} = 1/\tau$ and its optimum value is found using genetic algorithm^19^.

Also, from MKY Lemma^22^, $P$ is a symmetric positive definite matrix and $Q$ is a vector determined as

31 $$PA_{pe} + A_{pe}^{T} P = - Q,\;Q = Q^{T} > 0\;{\text{and}}\;PB_{1} = C_{pe}^{T} .$$

□

#### **Assumption 1**

The low-pass filtered parameter estimation error satisfies^20^,

32 $$\left\| {\overline{e}_{hf} } \right\| \le \omega \;\;{\text{and}}\;\;\left\| {\dot{\overline{e}}_{hf} } \right\| \le \upsilon ,$$

where ${ }\omega$, $\upsilon$ represents the respective positive upper bounds.

Now, taking time-derivative of the (28) we get

$$\dot{V}\left( {\overline{e},\tilde{\varphi }{ },\overline{e}_{hf} } \right) = 2\left( {{\Gamma }_{1} + {\Gamma }_{2} { }\overline{e}_{hf}^{T} {\Gamma }_{hf}^{ - 1} \overline{e}_{hf} } \right)\overline{e}^{T} P\dot{\overline{e}} + 2\left( {{{ \Gamma }}_{2} { }\overline{e}^{T} P\overline{e}{ }\overline{e}_{hf}^{T} {\Gamma }_{hf}^{ - 1} \dot{\overline{e}}_{hf} { }} \right) + 2\tilde{\varphi }^{T} \dot{\tilde{\varphi }} + 2{ }\overline{e}_{hf}^{T} {{ \Gamma }}_{hf}^{ - 1} \dot{\overline{e}}_{hf} .$$

From (22) and (29) substituting the value of $\dot{\overline{e}}$ and $\dot{\overline{e}}_{hf}$ in the above expression, considering $\Gamma \left( {\text{t}} \right) = \Gamma_{1} + \Gamma_{2} \overline{e}_{hf}^{T} \Gamma_{hf}^{ - 1} \overline{e}_{hf} ,$ we get

33 $$\begin{aligned} \dot{V}\left( {\overline{e},\tilde{\varphi }{ },\overline{e}_{hf} } \right) & = 2\Gamma \left( {\text{t}} \right){ }\overline{e}^{T} PA_{pe} \overline{e} + 2\Gamma \left( {\text{t}} \right)\overline{e}^{T} PB_{1} \tilde{\varphi }^{T} {\Phi } + 2{ }\overline{e}_{hf}^{T} \Gamma_{hf}^{ - 1} \dot{\overline{e}}_{hf} \left( {\Gamma_{2} { }\overline{e}^{T} P\overline{e} + 1} \right) + 2\tilde{\varphi }^{T} \dot{\tilde{\varphi }}, \\ \dot{V}\left( {\overline{e},\tilde{\varphi }{ },\overline{e}_{hf} } \right) & = \Gamma \left( {\text{t}} \right) \overline{e}^{T} \left( {A_{pe}^{T} P + A_{pe} P} \right)\overline{e} + 2\Gamma \left( {\text{t}} \right)\overline{e}^{T} PB_{1} \tilde{\varphi }^{T} {\Phi } + 2{ }\overline{e}_{hf}^{T} \Gamma_{hf}^{ - 1} \dot{\overline{e}}_{hf} \left( {\Gamma_{2} { }\overline{e}^{T} P\overline{e} + 1} \right) + 2\tilde{\varphi }^{T} \dot{\tilde{\varphi }}.{ } \\ \end{aligned}$$

From (31) the above expression can be written as

$$= - \Gamma \left( t \right) \overline{e}^{T} Q\overline{e} + 2 \Gamma \left( t \right)\overline{e}^{T} PB_{1} \tilde{\varphi }^{T} \Phi + 2 \overline{e}_{hf}^{T} \Gamma_{hf}^{ - 1} \dot{\overline{e}}_{hf} \left( {\Gamma_{2} \overline{e}^{T} P\overline{e} + 1} \right) + 2\tilde{\varphi }^{T} \dot{\tilde{\varphi }}.$$

From (25) substituting the value of $\dot{\tilde{\varphi }}$ in the above expression we get

$$= - \Gamma \left( t \right)\overline{e}^{T} Q\overline{e} + 2 \Gamma \left( t \right)\overline{e}^{T} PB_{1} \tilde{\varphi }^{T} \Phi - 2\Gamma \left( t \right)\tilde{\varphi }^{T} e_{o} \Phi + 2 \overline{e}_{hf}^{T} \Gamma_{hf}^{ - 1} \dot{\overline{e}}_{hf} \left( {\Gamma_{2} \overline{e}^{T} P\overline{e} + 1} \right).$$

Since, $PB_{1} = C_{pe}^{T}$ and it follows from the property 1 in^20,23^

$$2\Gamma \left( {\text{t}} \right)\tilde{\varphi }^{T} \left[ {\overline{e}^{T} e_{o} + Proj\left( {{{\varphi }}, - \overline{e}^{T} e_{o} } \right)} \right] \le 0.$$

Therefore,

$$\dot{V}\left( {\overline{e},\tilde{\varphi }{ },\overline{e}_{hf} } \right) = - {{ \Gamma }}\left( {\text{t}} \right)\overline{e}^{T} Q\overline{e} + 2{ }\overline{e}_{hf}^{T} {\Gamma }_{hf}^{ - 1} \dot{\overline{e}}_{hf} \left( {{\Gamma }_{2} { }\overline{e}^{T} P\overline{e} + 1} \right),$$

From (29) the above expression can be written as

$$\dot{V}\left( {\overline{e},\tilde{\varphi }{ },\overline{e}_{hf} } \right) = - { }\Gamma \left( {\text{t}} \right)\overline{e}^{T} Q\overline{e} + 2{ }\overline{e}_{hf}^{T} {\Gamma }_{hf}^{ - 1} \left( {\dot{\overline{e}} - {\Gamma }_{hf} \overline{e}_{hf} } \right)\left( {{\Gamma }_{2} { }\overline{e}^{T} P\overline{e} + 1} \right),$$

upon simplification, the above expression can be written as

$$\dot{V}\left( {\overline{e},\tilde{\varphi }{ },\overline{e}_{hf} } \right) = - {{ \Gamma }}\left( {\text{t}} \right)\overline{e}^{T} Q\overline{e} + 2{ }\overline{e}_{hf}^{T} \left( {{\Gamma }_{hf}^{ - 1} \dot{\overline{e}} - \overline{e}_{hf} } \right)\left( {{\Gamma }_{2} { } + 1} \right).$$

The above expression can also be written as

$$\dot{V}\left( {\overline{e},\tilde{\varphi }{ },\overline{e}_{hf} } \right) = - {{ \Gamma }}\left( {\text{t}} \right)\overline{e}^{T} Q\overline{e} + 2\frac{{{ }\overline{e}_{hf}^{T} }}{{{\Gamma }_{hf} }}\left( {\dot{\overline{e}} - {\Gamma }_{hf} \overline{e}_{hf} } \right)\left( {{\Gamma }_{2} { } + 1} \right),$$

from (29) the above expression can be expressed as

$$\dot{V}\left( {\overline{e},\tilde{\varphi }{ },\overline{e}_{hf} } \right) = - {{ \Gamma }}\left( {\text{t}} \right)\overline{e}^{T} Q\overline{e} + 2\frac{{{ }\overline{e}_{hf}^{T} }}{{{\Gamma }_{hf} }}\dot{\overline{e}}_{hf} \left( {{\Gamma }_{2} { } + 1} \right).$$

Now from Assumption 1, it follows

34 $$\dot{V}\left( {\overline{e},\tilde{\varphi }{ },\overline{e}_{hf} } \right) = - {{ \Gamma }}\left( {\text{t}} \right)\overline{e}^{T} Q\overline{e} + 2\frac{\omega }{{\left\| {{\Gamma }_{hf} } \right\|}}\upsilon \left( {{\Gamma }_{2} { } + 1} \right)$$

Now, it will be proved that for any $t > 0$, $V\left( {\tilde{\varphi },{ }\overline{e},\overline{e}_{hf} } \right) \le \chi$. Since $x\left( 0 \right) = x_{m} \left( 0 \right)$, therefore, it can be verified from (28) that ${ }V\left( 0 \right) \le \chi$, i.e.

$${ }V\left( 0 \right) \le \left\{ {4_{max} \left\| \varphi \right\|^{2} + 4_{max} \left\| {e_{hf} } \right\|^{2} } \right\} < \chi .$$

Now, in contradictory manner if we assume that there exists $t_{1} > 0$ such that $V\left( {t_{1} } \right) > \chi$, then from (28) we have

35 $${\Gamma }\left( {t_{1} } \right)\overline{e}^{T} \left( {t_{1} } \right)\overline{e}\left( {t_{1} } \right) > \frac{{2\lambda_{max} \left( P \right)\left[ {{\Gamma }_{2} + 1} \right]\omega \upsilon }}{{\left\| {{\Gamma }_{hf} } \right\|}}.$$

Since,

36 $$\begin{aligned} {\Gamma }\left( {t_{1} } \right)\overline{e}^{T} \left( {t_{1} } \right)\overline{e}\left( {t_{1} } \right) & \ge \frac{{{\Gamma }\left( {t_{1} } \right)\overline{e}^{T} \left( {t_{1} } \right)\overline{e}\left( {t_{1} } \right)}}{{\lambda_{max} \left( P \right)}} \\ & > \frac{{2\left[ {{\Gamma }_{2} + 1} \right]\omega \upsilon }}{{\left\| {{\Gamma }_{hf} } \right\|}} \\ \end{aligned}$$

Thus, from (34) and (36), it can be inferred that $\dot{V}\left( {t_{1} } \right) < 0$. Therefore, $V\left( t \right){ }$ will always lie in the set $\Omega_{R} = \left\{ {V\left( t \right):V\left( t \right) \le \chi } \right\}$. Hence, it can be concluded that $V\left( t \right) \le \chi ,$ for any $t \ge 0$. Since,

37 $$\lambda_{min} \left( P \right)\left\| {\overline{e}} \right\|_{2}^{2} \left( {{\Gamma }_{1} + {\Gamma }_{2} { }\overline{e}_{hf}^{T} {{ \Gamma }}_{hf}^{ - 1} \overline{e}_{hf} } \right) \le \left( {{\Gamma }_{1} + {\Gamma }_{2} { }\overline{e}_{hf}^{T} {{ \Gamma }}_{hf}^{ - 1} \overline{e}_{hf} } \right)\overline{e}^{T} P\overline{e} \le V\left( t \right).$$

Now from the above expression (37) it can be inferred that

38 $$\lambda_{min} \left( P \right)\left\| {\overline{e}} \right\|_{2}^{2} \left( {{\Gamma }_{1} + {\Gamma }_{2} \omega^{2} { }\left\| {{\Gamma }_{hf}^{ - 1} } \right\|} \right) \le \chi ,$$

this implies

$${ }\left\| {\overline{e}} \right\|_{2} \le \sqrt {\frac{\chi }{{\lambda_{min} \left( P \right)\left( {{\Gamma }_{1} + {\Gamma }_{2} \omega^{2} { }\left\| {{\Gamma }_{hf}^{ - 1} } \right\|} \right)}}} .$$

Since, from^16,20,24^, the inequality follows $\left\| {\overline{e}} \right\|_{\infty } \le \left\| {\overline{e}} \right\|_{2}$ holds therefore, the results in (28) immediately follows. Similarly,

$$\begin{aligned} \left\| {\tilde{\varphi }} \right\|_{\infty } & \le \chi ,\;\;{\text{and}} \\ \left\| {\overline{e}_{hf} } \right\|_{\infty } & \le \chi . \\ \end{aligned}$$

Therefore, $\left( {\overline{e},\tilde{\varphi },\overline{e}_{hf} } \right)$ is bounded.

The overall derived HFLAG-MRAC rules can be summarized as follows:

39 $$\begin{aligned} \dot{\delta }_{1} & = - \mu \delta_{1} + u_{p} , \\ \dot{\delta }_{2} & = - \mu \delta_{2} + y_{p} , \\ {\dot{\Phi }} & = - \alpha {\Phi } + \delta ,\quad \delta = \left[ {r,\delta_{1} ,\delta_{2} ,y_{p} } \right]{ }^{T} , \\ u_{p} & = \varphi^{T} \delta + \dot{\varphi }^{T} {\Phi } = \varphi^{T} \delta - \Phi^{T} {\Gamma }\left( {\text{t}} \right){ }Proj{ }\left( {\varphi ,e_{0} {\Phi }} \right), \\ \dot{\varphi } & = - \left( {{\Gamma }_{1} + {\Gamma }_{2} { }\overline{e}_{hf}^{T} {{ \Gamma }}_{hf}^{ - 1} \overline{e}_{hf} } \right){ }Proj{ }\left( {\varphi ,e_{0} {\Phi }} \right). \\ \end{aligned}$$

The update laws for proposed HFLAG-MRAC controller mentioned in (39) can be interpreted as, the proposed controller has two distinct gain parameters terms for adjustment. First term is static gain term ${\Gamma }_{1}$, which should be less for ideal controller design. It is the gain when error ceases to zero i.e., the static gain ${\Gamma }_{1}$ is the minimum gain required for controller to under no or small variations in input. The second term ${\Gamma }_{2}$ is the adjustable gain term, which is a function of high-frequency content of the tracking (output) error. In other words, ${\Gamma }_{2}$ is adjusted in response to the variations in environmental conditions i.e., rapidly changing external input perturbations.

To obtain the high-frequency or ripple content of tracking error, the tracking (output) error is passed through low-pass filter with optimum value of τ, and this low pass filtered version of error is then subtracted from the original tracking error. The adaptation gain is adjusted according to the resultant high-frequency or ripple content of the tracking which ensure the absenteeism of high-frequency oscillations in control signal. Thus, we propose the name High-Frequency Learning based Adjustable Gain-MRAC (HFLAG-MRAC). The proposed HFLAG-MRAC controller guarantees fast convergence, improved transient performance and overall stability without the need of high adaptation gain.

## Simulation model of proposed 2-level MPPT control

**Figure 10:**
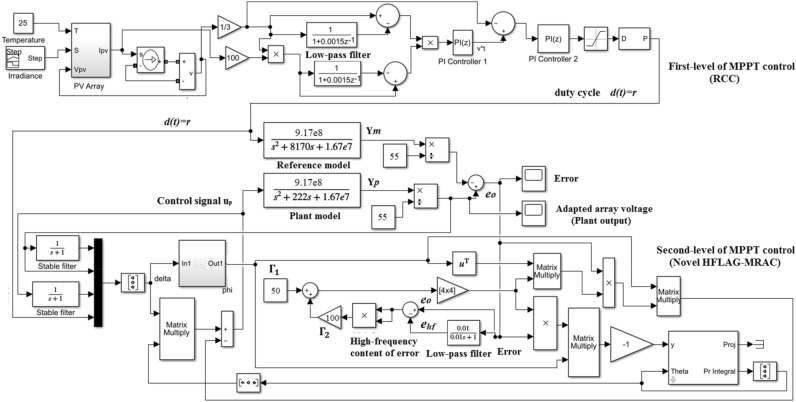
Simulink model of proposed 2-level MPPT control.

For verification of the proposed HFLAG-MRAC adaptive controller for 2-level MPPT control, a simulation model has been developed in Simulink as shown in Fig. 10. RCC takes inputs from PV panel and calculates duty cycle $d\left( t \right) = r$, input for the developed HFLAG-MRAC level, which is designed using the law mentioned in (39). The plant, and the reference model parameters are considered as shown in Table 2.

**Table 2:** Simulation parameters for the proposed HFLAG-MRAC.

Parameter	Value
$R$	17 Ω
$L$	600 μH
$C$	100 μF
$V_{o}$	55 V
$k_{p} = V_{o} /LC$	9.17 × 10^8^ V (rad/s)^2^
$a_{p} = 1/RC$	1500 rad/sec (ζ = 0.18)
$b_{p} = 1/LC$	1.67 × 10^7^ (rad/s)^2^
$k_{m}$	9.17 × 10^8^ V (rad/s)^2^
$a_{m}$	8.17 × 10^3^ (rad/s)
$b_{m}$	1.67 × 10^7^ (rad/s)^2^
μ, α	1
${\Gamma }_{1}$	50
${\Gamma }_{2}$	100

The plant model is opted such that, it will exhibits actual array voltage output for an underdamped step response with ζ = 0.18. The reference model is obtained from the underdamped boost converter plant model, by making ζ of (10) equal to 1. For simplification in analysis, scale of the array voltage $V_{o}$ has been normalized from 0–55 V to 0–1 V. The performance of the proposed 2-level MPPT with HFLAG-MRAC has been compared with the 2-level MPPT scheme with conventional high-static gain MRAC^2^.

## Simulation results and discussions

### Under uniform solar insolation condition

**Figure 11:**
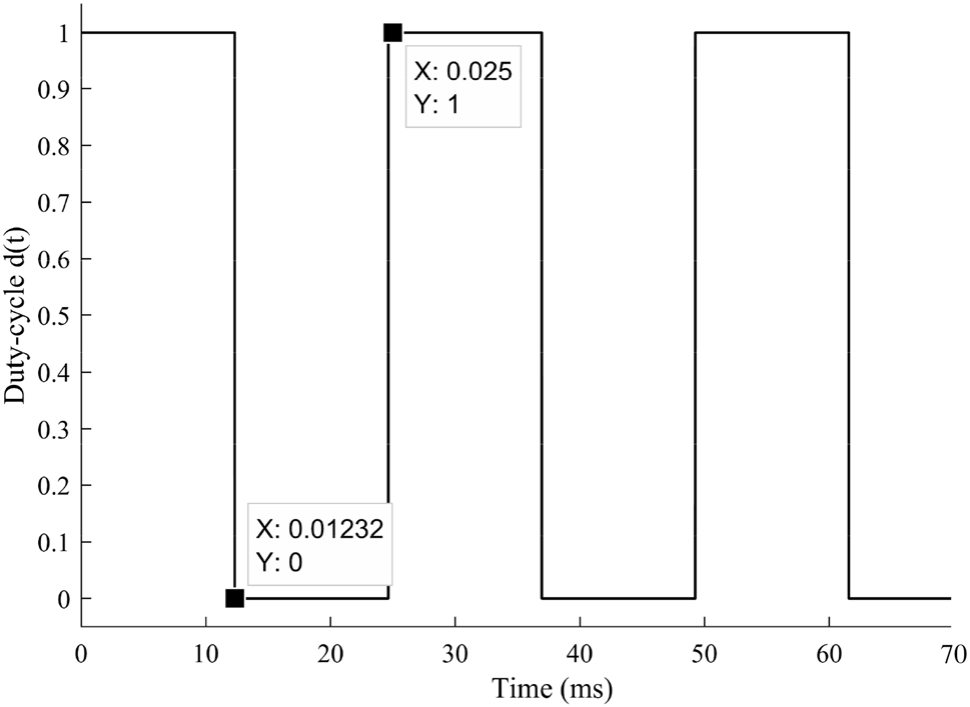
PWM duty-cycle $d\left( t \right)$, output of RCC unit.

The duty-cycle output $d\left( t \right)$, which serves as an input for HFLAG-MRAC control level, is shown in Fig. 11. It is the output of the RCC unit, calculated using array voltage and power inputs. The squared pulse width modulated (PWM) signal output of RCC, represents the changes occurs in solar insolation, from 1 to 0 and 0 to 1 at 12.3 ms and 25 ms respectively. Under uniform solar insolation condition, the duty cycle on-time remains same.

**Figure 12:**
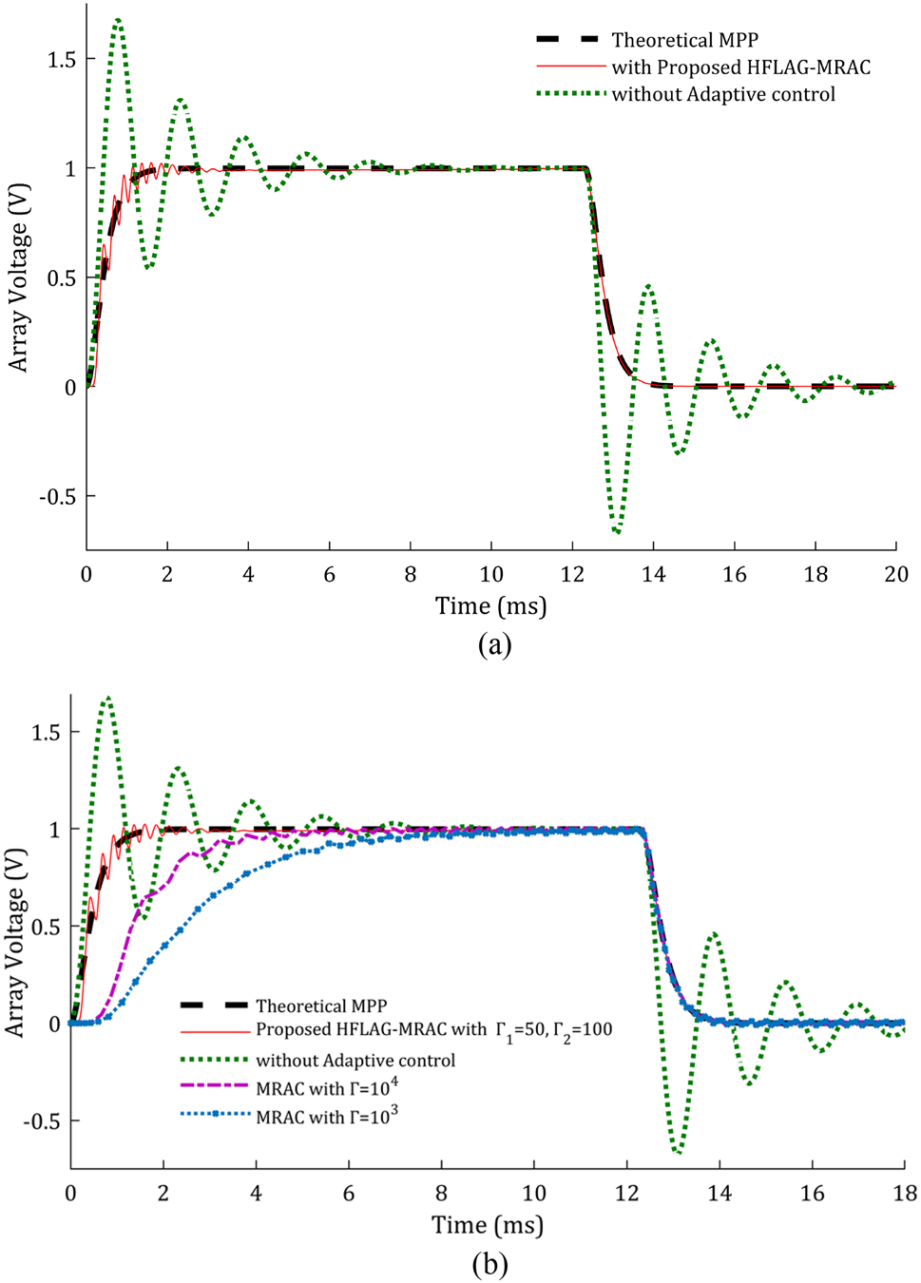
(**a**) Performance of adapted array voltage with the proposed HFLAG-MRAC, theoretical MPP voltage and un-adapted array voltage at early adaptation stage, (**b**) Array voltage performance comparison at early adaptation stage.

Performance comparison of the adapted array voltage with proposed 2-level MPPT using HFLAG-MRAC has been done with theoretical MPP, without adaptive control and 2-level MPPT using MRAC^2^ and shown in Fig. 12a and b. To check the performance of the proposed scheme, the control intervals are divided into 2 stages, early adaptation and late adaptation stage. Figure 12a and b, represent the early adaptation stage of array voltage, in which the output array voltage along with theoretical MPP voltage and unadapted array voltage are shown. The fast convergence of proposed scheme can be seen from Fig. 12a and b. A comparison at early adaptation stage of proposed scheme without adaptive control and with conventional MRAC MPPT^2^ for different gain values is presented in Fig. 12b.

It can be observed that the adapted array voltage of proposed approach converges to the theoretical MPP voltage faster as compared to the conventional MRAC MPPT cases. Also, it can be seen that with the novel HFLAG-MRAC the plant starts to learn within 1 ms, and the adapted array voltage begins to dampen just after 2 ms. Whereas, even with very high gain, the plant adapts theoretical MPP after 4 ms for Г = 10^4^ and relatively late for the gain of Г = 10^3^ for conventional MRAC cases. The unadapted array voltage without any adaptive control scheme, continues to oscillate and reach the theoretical steady-state MPP voltage after 8 ms. Eventually, this reveals the precision of RCC to estimate the accurate optimal duty cycle which can deliver maximum available power in the steady state. At 12.3 ms, solar insolation changes and unadapted array voltage shows an underdamped response again; whereas, the proposed controller only needs initial learning to continually exhibit a critically damped characteristic.

**Figure 13:**
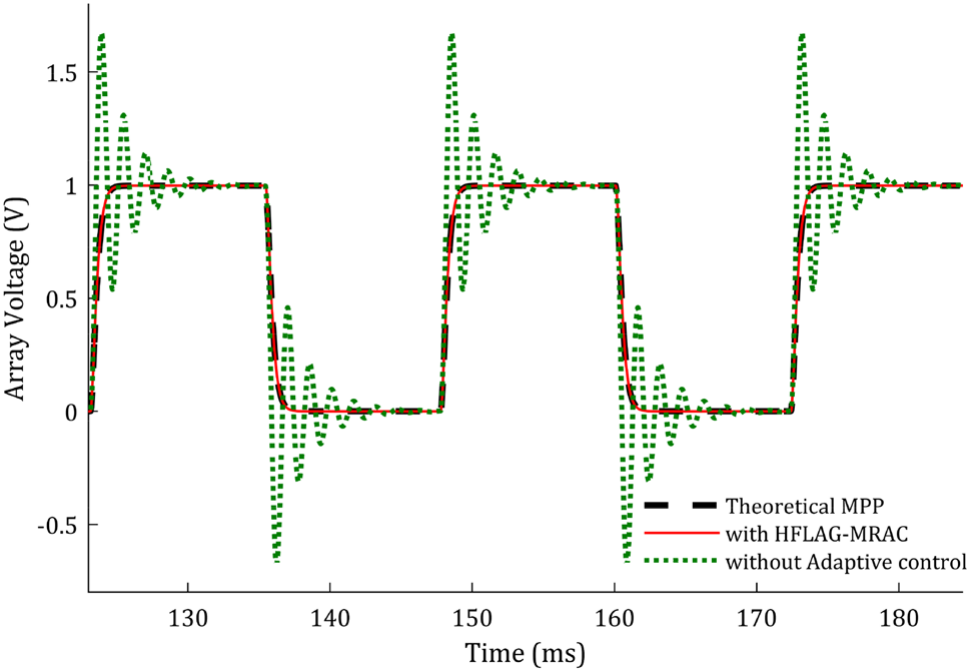
Performance of the theoretical MPP voltage, un-adapted array voltage and the proposed HFLAG-MRAC at late adaptation stage.

Figure 13, represents the un-adapted array voltage and adapted array voltage response for proposed scheme for later adaptation stage with theoretical MPP voltage. In the later stage of adaptation, the adapted array voltage has almost completely converged to the theoretical MPP, and during changes in solar insolation, no fluctuations can be seen in it. Elimination of such fluctuations in plant’s underdamped response under rapidly changing solar insolation conditions using HFLAG-MRAC, is one of the objectives of this work. It can be also confirmed from the Figs. 1 and 6 that during changes in solar insolation, the unadapted array voltage without MPPT fluctuates to the left and right of the MPP, before finally reaching the MPP, which can be verified from the simulation studies. Whereas, the adapted array voltage converges directly to the theoretical MPP with no fluctuations, just after initial learning phase. At every transition points the proposed HFLAG-MRAC adaptive controller just adjusts its gain to minimize the tracking error and the adapted array voltage follows theoretical MPP voltage, without any fluctuations.

**Figure 14:**
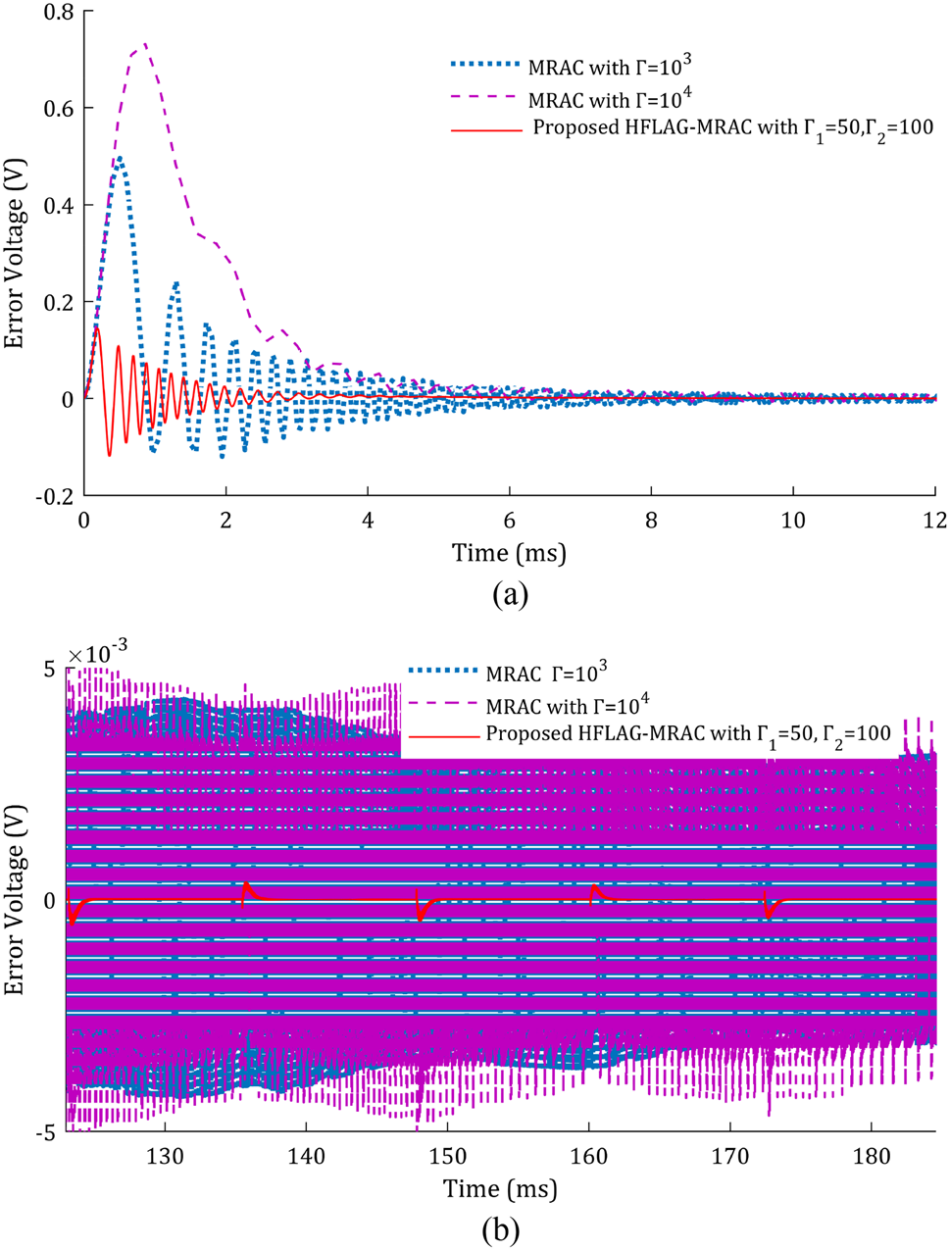
(**a**) Comparison of error voltage at early adaptation stage, (**b**) Comparison of error voltage at later adaptation stage.

The error voltage (tracking error) comparison at early and later adaptation stages have been shown in Fig. 14a and b. It can be seen that the tracking error of conventional MRAC MPPT has high-frequency oscillations, in both early and late stages of adaptation. As shown in Fig. 14b, it can be observed that even after completion of learning phase, the late adaptation error voltage of MRAC MPPT for Г = 10^3^ are having high-frequency oscillations and increasing for higher values of gain e.g., Г = 10^4^. These high-frequency oscillations in error may cause numerical instability and make the adaptive law of the controller stiff, as the adaptive law is error dependent. Whereas, the error voltage using HFLAG-MRAC controller has the absence of such high-frequency oscillations in early as well as later adaptation stages. As discussed previously, in the proposed MPPT control the HFLAG-MRAC level is developed to learn from the high-frequency content of tracking error, to avoid appearance of detrimental high-frequency oscillations at any phase of the controller design, which can be verified from the Fig. 14a and b. With HFLAG-MRAC system learns from the high-frequency content of error and after initial learning, the error converges asymptotically to zero, as proved mathematically in “The proposed HFLAG-MRAC controller” section, and can be verified from the simulation results as shown in Fig. 14a and b.

**Figure 15:**
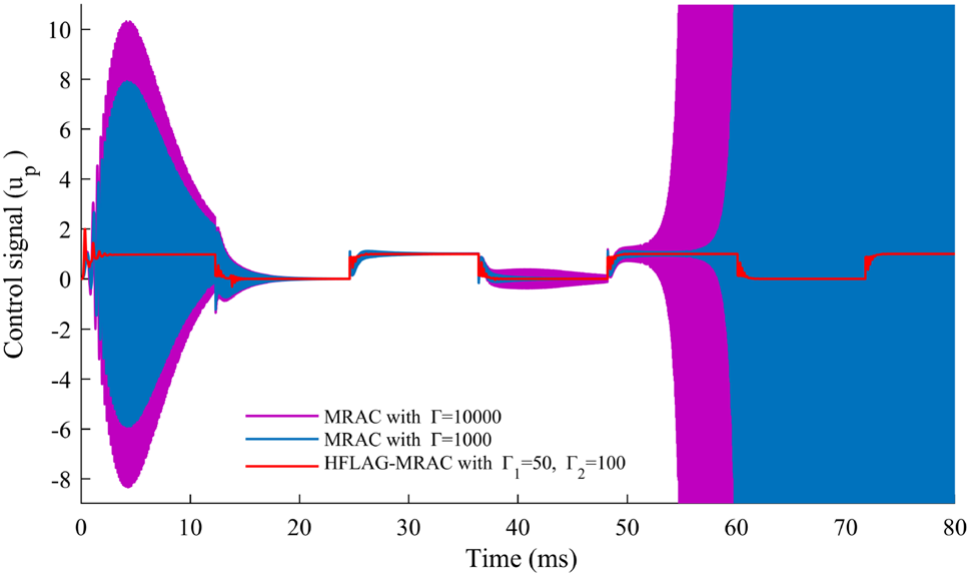
Comparison of control signals $u_{p}$.

In Fig. 15 an important aspect of adaptive controller design for any application has been shown. After verification of the adapted array voltage and error voltage, performance of the proposed HFLAG-MRAC controller has been tested and compared with MRAC, for the quality of control signal. The control signal of the controller must not have high-frequency oscillations. These high-frequency oscillations may cause numerical instability and make the adaptive law stiff, which in turn may leads system to instability^15,16,20^.

A comparison of control signals of HFLAG-MRAC with MRAC^2^ has been presented in Fig. 15. It can be clearly observed that even after initial learning phase, the control signal of conventional MRAC has continuous presence of high-frequency oscillations in its control signal in both the gain cases. Also, for higher gain values the content of high-frequency oscillations in the control signal increased more, which might mean that the signal does not have any information^16,17,20^. Whereas, the control signal of developed HFLAG-MRAC is free from such oscillations. After initial learning the proposed HFLAG-MRAC adjusts its gain at transition points and remains stable otherwise. Actually, these are the transition points where solar insolation changes and controller adjusts its gain accordingly to these variations to minimize the tracking error.

### Under partly-cloudy weather conditions

**Figure 16:**
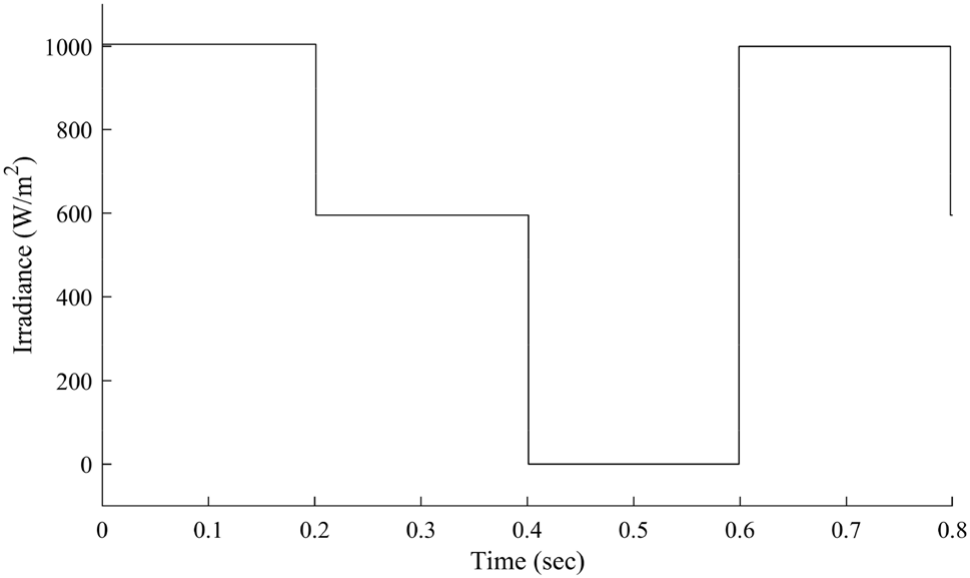
Step input irradiance representing varying solar insolation.

**Figure 17:**
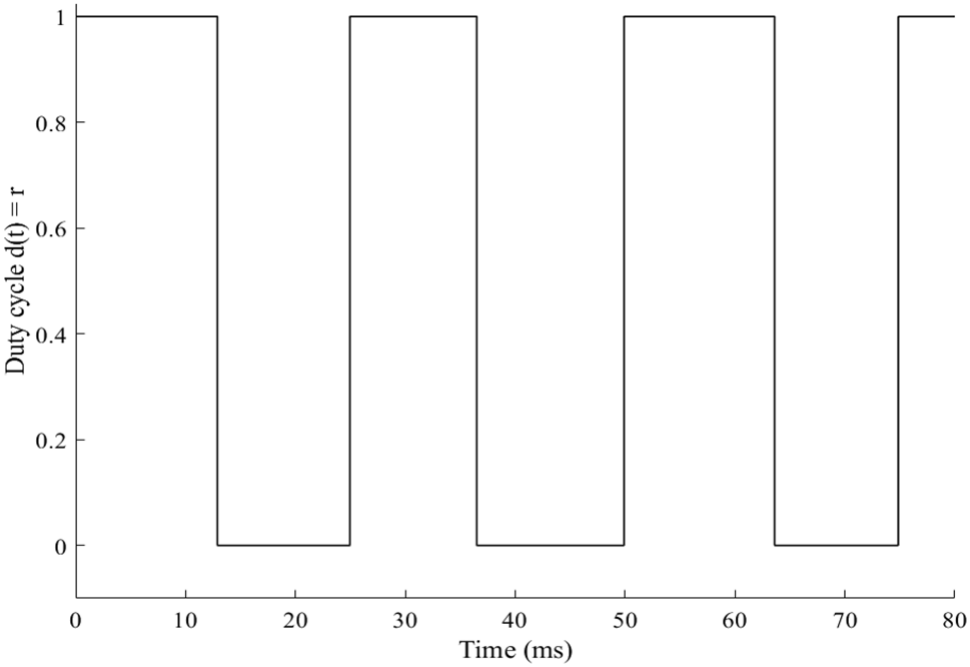
Duty cycle update for varying solar insolation.

**Figure 18:**
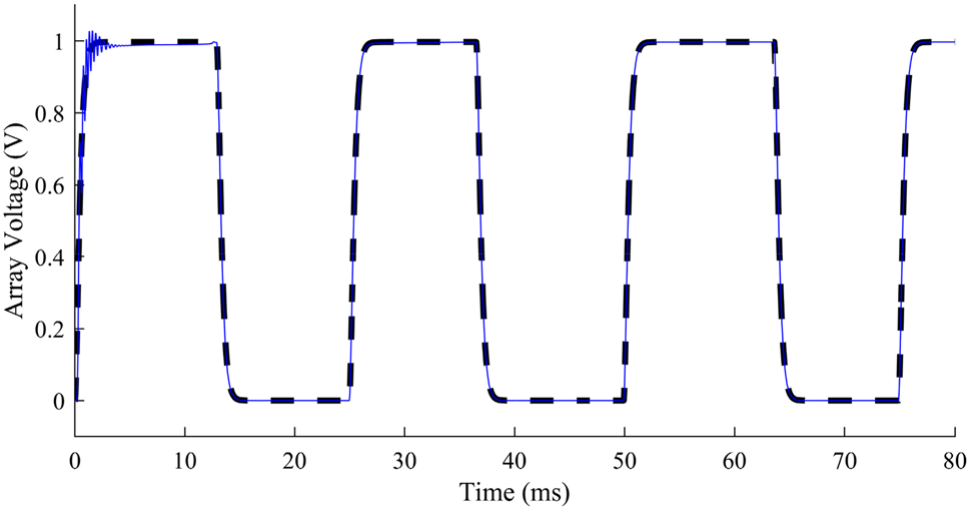
Output performance of the proposed HFLAG-MRAC under varying solar insolation.

**Figure 19:**
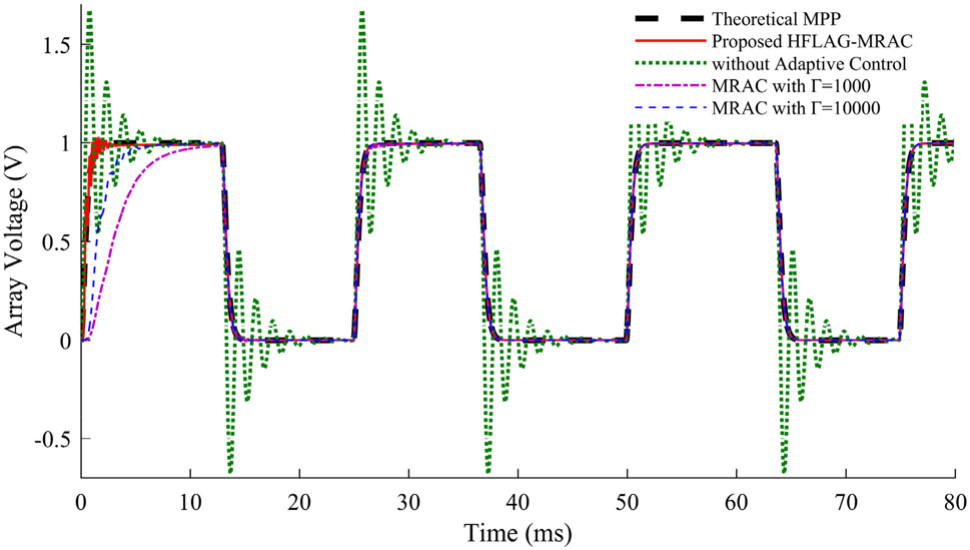
Output comparison of the proposed HFLAG-MRAC with other control schemes under varying solar insolation.

**Figure 20:**
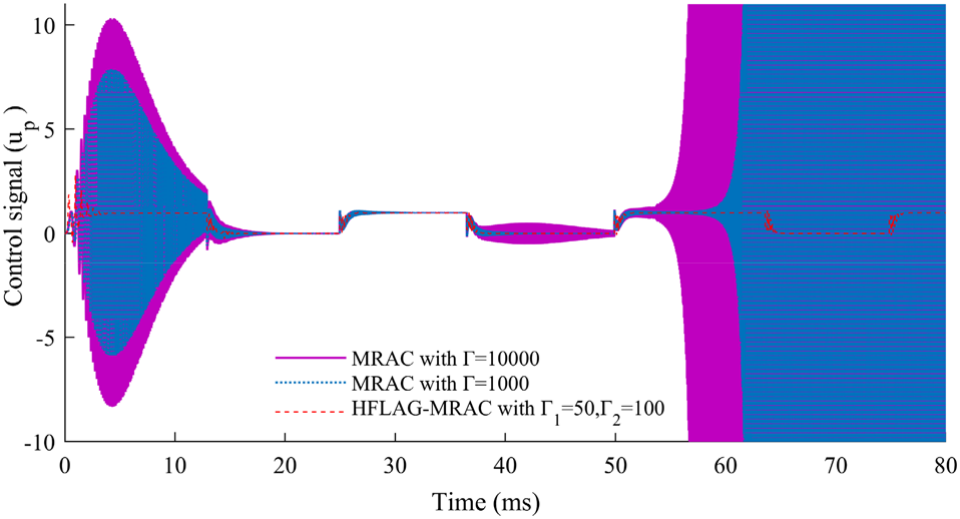
Control signal comparison of the proposed HFLAG-MRAC with other control schemes under varying solar insolation.

The step input solar irradiance profile representing cloudy weather conditions is shown in Fig. 16. The simulations under different irradiance conditions are performed and the corresponding updates in the duty cycle are shown in Fig. 17. Figure 17 shows the corresponding updates in the on-time of RCC's duty cycle in response to the changing solar irradiance (shown in Fig. 16). The output performance of the proposed HFLAG-MRAC is shown in Fig. 18, and the performance comparisons for output array voltage and control signal with the other control schemes are shown in Figs. 19 and 20.

## Experimental results

**Figure 21:**
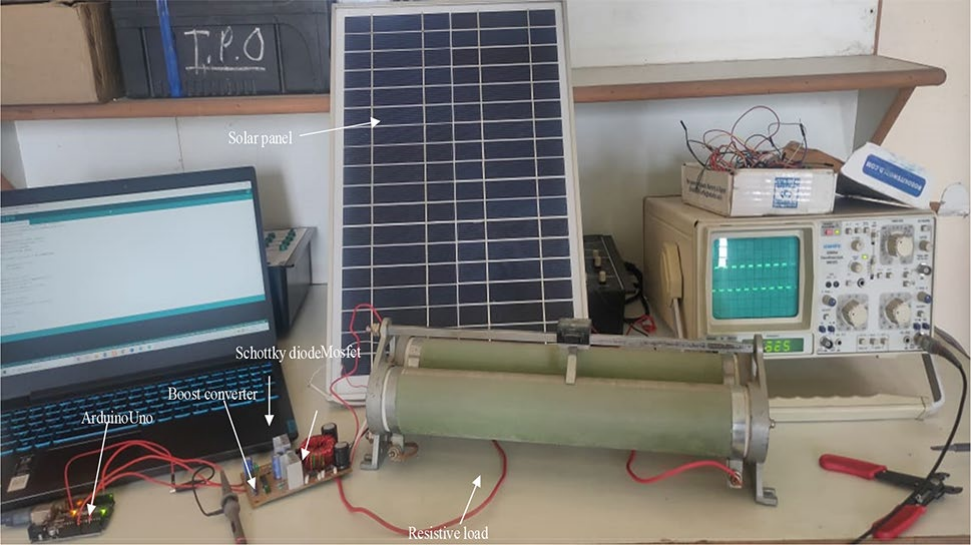
Experimental setup for validation.

**Figure 22:**
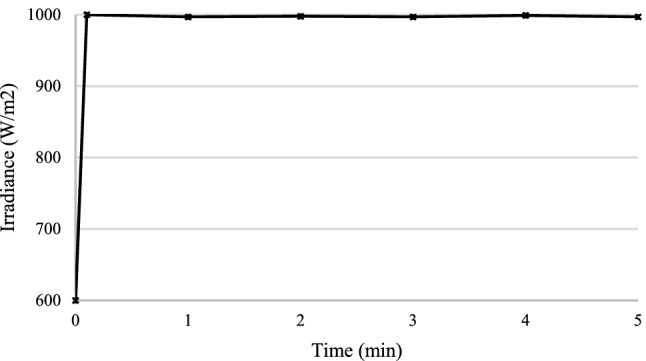
Measured uniform solar irradiance profile.

**Figure 23:**
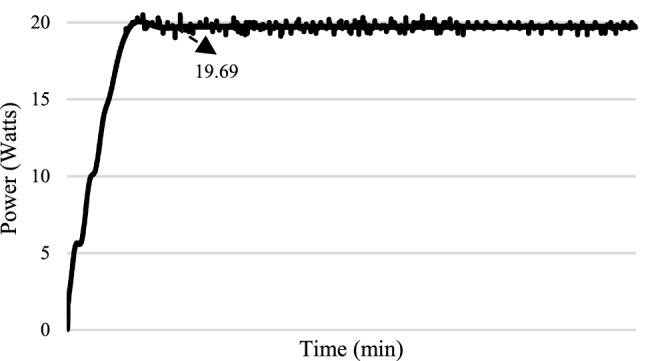
Output power of the proposed MPPT scheme corresponding to uniform solar irradiance.

**Figure 24:**
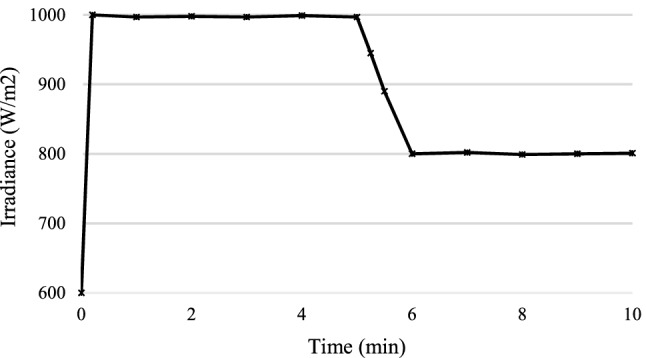
Measured solar irradiance profile under cloudy weather conditions.

**Figure 25:**
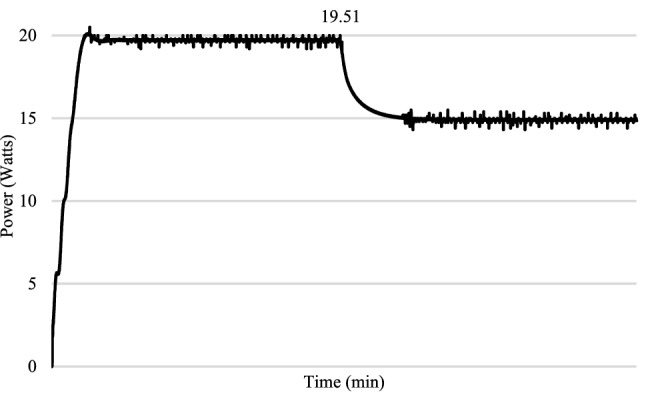
Output power of the proposed MPPT scheme under varying solar insolation.

For the experimental validation, a hardware prototype has been developed (see Fig. 21). Table 3 shows the hardware specifications of the developed prototype followed by PV panel specifications in Table 4 and boost converter specifications in Table 5. The entire code for RCC and HFLAG-MRAC is converted in embedded C using MATLAB embedded coder. Figures 22, 23, 24 and 25 shows the experimental outcomes of the proposed system under uniform and varying solar irradiance conditions. The experiments are conducted on a cloudy weather day, where the readings of the measured solar irradiance during the initial 5 min interval is maintained around 1000 W/m^2^ (see Fig. 22). The corresponding output power is shown in Fig. 23. The solar irradiance readings after 5 min reduced around 800 W/m^2^, due to the presence of clouds (see Fig. 24). The corresponding output power is shown in Fig. 25, in which it can be seen that output is free from transient as well as steady-state oscillations.

**Table 3:** Hardware specifications.

S. no	Component name	Specifications
1	Schottky diode MBR20100	20 A, 100A peak reverse voltage
2	Toroidal core T-45	Size: Outside Dia = 45.4 mm (1.79″), Inner Dia = 27.1 mm (1.06″), Height = 11.5 mm (0.45″)
3	Mosfet IRFZ44N	$V_{DSS} = 55\;{\text{V}}$, I_D_ = 49A,$V_{{DS\left( {on} \right)}} = 7.5\;{\text{m}}\Omega$
4	Gate Driver TL494	Integrated error amplifier with inbuilt oscillator
5	Arduino Uno	ATMega328, 6 channels 10 bit ADC, 6 PWM channels
6	Hall effect current sensor WCS1700	Zero $V_{out} = 2.5\;{\text{V}}$, Sensitivity = 33 mV/A Bandwidth = 23 kHz
7	Loom Solar panel	Peak power = 20 W, $V_{oc} = 21\;{\text{V}}$, $I_{sc} = 1.27\;{\text{A}}$
8	CRO (Scientific)	2 channel, operating frequency 25 MHz
9	Capacitor	100 uf, 50 v
10	Toroidal inductor	600 μH

**Table 4:** PV panel specifications.

PV module specifications	Values
No. of series connected strings	1
No. of parallel connected strings	1
Open circuit voltage $V_{oc}$ of the PV module	21 (V)
Short circuit current $I_{sc}$	1.27 (A)
MPP voltage $V_{M}$	17.4 (V)
MPP Current $I_{M}$	1.18 (A)
Maximum Power $P$	20 (W)
Maximum system voltage	1000 (V)

**Table 5:** Boost converter specifications.

Parameter specifications	Values
PV module resistance $R$	17 Ω
Inductor $L$	600 μH
Capacitor $C$	100 μF
Input to the boost converter	16.8 (V)
Output voltage $V_{o}$ of the plant	55 (V)
DC link capacitor	100 μF
Load resistance (for experiment)	300 Ω

The performance comparison analysis in terms of power, tracking efficiency, and convergence speed under uniform and variable solar irradiance is presented in Tables 6, 7 and 8. In Table 5, it can be seen that the power efficiency of the proposed HFLAG-MRAC based MPPT scheme is more compared to the MRAC based MPPT. Moreover, the superiority of the proposed MPPT scheme in terms of tracking efficiency and convergence speed can be seen in Tables 7 and 8.

**Table 6:** Power efficiency under different environmental scenarios.

Scenario	Power efficiency	
	HFLAG-MRAC (%)	Conventional MRAC (%)
Under constant solar insolation	98.45	96.50
Under changing solar insolation (partly cloudy conditions)	97.55	94.35

**Table 7:** Tracking efficiency under different environmental scenarios.

Scenario	Tracking efficiency	
	HFLAG-MRAC (%)	Conventional MRAC (%)
Under constant solar insolation	98.65	96.25
Under changing solar insolation (partly cloudy conditions)	98.25	95.60

**Table 8:** Convergence Speed under different environmental scenarios.

Scenario	Convergence speed	
	HFLAG-MRAC (ms)	Conventional MRAC (ms)
Under constant solar insolation	2.1	4.2
Under changing solar insolation (partly cloudy conditions)	2.3	4.7

## Conclusion

In this work, a 2-level MPPT control architecture has been proposed to achieve fast convergence with improved steady-state and transient performance under varying environmental conditions. The conventional RCC MPPT is used to a yield ripple-free optimum duty cycle in the steady-state at the first level, which acts as an input to the novel HFLAG-MRAC at the second level. In the proposed HFLAG-MRAC, the adaptation gain is adjusted as a function of the high-frequency (ripple) content of tracking error and ensures absenteeism of high-frequency oscillations in the control signal. Thus guaranteed transient response, fast convergence and overall system stability are ensured, without needing high adaptation gain. Simulation studies and experimental analysis confirms the superiority of the proposed work over other 2-level MPPT control schemes.
